# Experimental and computational analysis of glycan processing at increased monoclonal antibody productivities

**DOI:** 10.1016/j.mec.2026.e00285

**Published:** 2026-07-20

**Authors:** Ranya Pranomphon, Sofia Gialamoidou, Montarop Yamabhai, Susan T. Sharfstein, Ioscani Jiménez del Val

**Affiliations:** a College of Nanotechnology, Science and Engineering and The RNA Institute, University at Albany, State University of New York, Albany, NY, 12203, USA; b School of Biotechnology, Institute of Agricultural Technology, Suranaree University of Technology, Nakhon Ratchasima, Thailand; c School of Chemical & Bioprocess Engineering, University College Dublin, Dublin, D04 V1W8, Ireland

**Keywords:** Monoclonal antibody, Adalimumab, Bioprocess conditions, Glycosylation, CHO cells

## Abstract

Optimization of cell culture conditions is critically important for successful commercial production of therapeutic antibodies; however, improvements in productivity may be offset by changes in critical quality attributes. We assessed the impact of two different process conditions (high and low yield) on growth, productivity, and glycan distribution for two CHO-S clones producing adalimumab, clone 1 (DHFR clone) and clone 2 (GS clone) in fed-batch shake flask cultures. High-yield cultures showed approximately 1.6-fold and 7.4-fold increases in integral of viable cells (IVC) for clones 1 and 2, respectively, compared to the low-yield cultures. Although specific productivity was lower in the high-yield cultures, this decrease was offset by the increased IVC, yielding higher titers in both cell lines. High-yield cultures showed a marked decrease in galactosylation, marked by a decrease in G1F glycans compared with the low-yield cultures, across both clones. Man5 glycans remained unchanged in clone 1 cultures, while a slight increase was observed in the high-yield clone 2 cultures. Metabolic analysis showed significant changes in glucose utilization in high-yield cultures with substantial decreases in specific glucose uptake and lactate yield, particularly for clone 2. Calculation of nucleotide sugar fluxes revealed that the increased utilization of UDP-N-acetylglucosamine and UDP-galactose for cellular glycosylation limited their availability for monoclonal antibody (mAb) glycosylation in high IVC, high-yield cultures, consistent with the observed changes in mAb glycans.

## Highlights

• Increased agitation enhances CHO cell growth and mAb titer via enhanced oxidative metabolism.
• High-yield cultures shift mAb Fc glycosylation towards less processed glycoforms.
• Stoichiometric NSD demand framework identifies drivers of mAb glycosylation shifts.
• IVC is the strongest predictor of mAb glycan processing across culture conditions.

## 1 Introduction

Monoclonal antibodies (mAbs) have dominated therapeutic proteins, providing new treatments for a wide range of conditions and indications. The global therapeutic mAb market was estimated at approximately $227 billion in 2023, with a projected compound annual growth rate of about 12% from 2024 to 2033 (https://www.precedenceresearch.com/monoclonal-antibodies-market).

The expiration of patents and data protection periods for innovator products, particularly blockbuster monoclonal antibodies (mAbs), e.g., adalimumab (Humira), trastuzumab (Herceptin), and bevacizumab (Avastin), creates opportunities for other manufacturers to develop biosimilar versions at reduced costs (Pranomphon et al., 2024). This drives down drug prices and expands the availability of these molecules to low and middle-income countries. Reducing the manufacturing costs of biosimilar products is also essential to ensure market penetration (Blackstone and Joseph, 2013).

Biosimilars must be highly similar to an already authorized originator biologic product in terms of their physicochemical and biological properties, with no meaningful clinical differences (Hajba et al., 2018). The majority of these therapeutic antibodies are produced in mammalian cell cultures, with Chinese hamster ovary (CHO) cell systems being the preferred host organism due to their human-like glycosylation, high productivity, and ability to grow at high densities for large-scale production (Kavoni et al., 2025; Muralidharan-Chari et al., 2021).

Adalimumab (Humira) is a fully human monoclonal antibody of the IgG1 subclass. It specifically binds to the proinflammatory cytokine, tumor necrosis factor alpha (TNFα), and neutralizes its biological activity by binding to both soluble and transmembrane forms of TNF, thereby inhibiting the interaction of TNF with p55 and p75 cell-surface TNF receptors (Alsamil et al., 2021; Markus et al., 2019). Adalimumab was approved by the FDA (U.S. Food and Drug Administration) in 2002 for the treatment of moderate-to-severe rheumatoid arthritis (Bang and Keating, 2004; Gao et al., 2022; Scheinfeld, 2003). Since then, it has been approved for other indications, including juvenile idiopathic arthritis, psoriatic arthritis, ankylosing spondylitis, Crohn's disease, and plaque psoriasis (Bandyopadhyay et al., 2014; Millán-Martín et al., 2023).

Glycosylation is one of the most important critical quality attributes in biosimilar development, as it significantly impacts the drug's biological activity, stability, immunogenicity, serum half-life, and effector functions (Cobb, 2020; Lakshmanan et al., 2024). The IgG-Fc region contains a conserved N-linked glycosylation site (Asn297), where oligosaccharides are covalently attached to an asparagine residue in the heavy chain constant domain 2 (CH2) (Rameez et al., 2021; Wei et al., 2021).

Controlling glycosylation is challenging because glycan synthesis is a non-templated process. It can be influenced by various factors, including host cell types, glycoengineering, and bioprocess optimizations such as nutrient availability, dissolved oxygen levels, media components, vessel types, temperature, pH, operation schedules, and culture modes (Madabhushi et al., 2021; Pranomphon et al., 2024; Zhang et al., 2016). Shifts in glycosylation can be caused by glycosylation enzyme availability/activity relative to specific productivity (Jimenez del Val et al., 2016a) and/or fluctuations in nucleotide sugar donor (NSD) availability (Chee Furng Wong et al., 2005; Kochanowski et al., 2008; Nyberg et al., 1999; Wong et al., 2010).

Two adalimumab-producing CHO clones were established and grown in fed-batch shake flask cultures. The agitation rate of the fed-batch shake flask cultures was increased to enhance product yield. Although this simple modification substantially increased cell growth and mAb titer, it also altered metabolic and glycan profiles – particularly mAb Fc β4-galactosylation. To identify the cause of these excursions, we further characterized cellular metabolism and quantified NSD consumption.

Insights into the mechanisms whereby higher agitation increased cell growth and mAb yield were obtained by comparing the computed glucose uptake and lactate production rates for both clones cultured under low- and high-yield conditions. To investigate why galactosylated species decreased in the high-yield cultures, the rate of uridine diphosphate galactose (UDP-Gal) consumption towards cellular and mAb N-linked glycosylation was computed with experimental data and a theoretical framework that uses stoichiometric coefficients for the composition of cellular N-glycans (Jimenez del Val et al., 2016b).

## 2 Materials and methods

### 2.1 Cell lines

Two different adalimumab-producing CHO cell lines, generated with different selection technologies, were used in this study. For clone 1, a vector containing dihydrofolate reductase (DHFR) and puromycin resistance genes was transfected into CHO-S cells, which were then selected with methotrexate (MTX) and puromycin, as described previously (Jarusintanakorn et al., 2021). Clone 2 was developed by transfecting an adalimumab expression vector containing a glutamine synthetase (GS) gene into single GS-knockout CHO-S cell line (a CHO-S clone with deletion of a single GS5 allele using CRISPR/Cpf1). These cells were then selected using methionine sulfoximine (MSX), as described previously (Srila et al., 2023). Cell lines were expanded for at least two passages after thawing before experiments were performed. For clone 1, cells were routinely cultured in Gibco™ Dynamis™ Medium (Thermo Fisher Scientific) supplemented with 8 mM GlutaMAX (Thermo Fisher Scientific) and 1% anti-clumping agent (Thermo Fisher Scientific), while for clone 2, cells were cultured in Dynamis medium supplemented with 0.2% anti-clumping agent, without GlutaMAX. Cultures were maintained on an orbital shaker at 37 °C, 130 rpm, and 7% CO_2_.

### 2.2 Fed-batch shake flask cultures

Clone 1 and clone 2 were grown in fed-batch shake flask cultures under two different conditions, low yield and high yield. In both conditions, cells were seeded at 0.3 × 10^6^ cells/mL in duplicate 125 mL shake flasks (Thomson Optimum Growth® Flasks or Nest Scientific) with a 30 mL working volume in Dynamis media as described above. Cells were fed with Gibco™ EfficientFeed™ C + AGT™ supplement (Thermo Fisher Scientific), a concentrated, proprietary nutrient mix, daily, starting on day 2. The feed supplement, at a 2x concentration, was added at 1.9% of the initial culture volume. Additional glucose was added when the glucose level fell below 6 g/L in the culture medium. Cultures were harvested when viability dropped to ∼60%.

In the low-yield condition, the agitation speed was maintained at 130 rpm for both clones. In the high-yield conditions, agitation speed was increased to 150 rpm for both clones. For clone 1, an additional 2 mM GlutaMAX was added on day 5 to prevent glutamine depletion. In addition, the anti-clumping agent concentration was increased to 1% for clone 2.

### 2.3 Cell counts, cell size, extracellular metabolites, and osmolality

Viable cell density and cell diameter were measured by trypan blue dye exclusion using either an automated Luna II® cell counter (Logos Biosystems) or a TC10™ cell counter (Bio-Rad). Cell-culture samples (500 μl) were collected daily prior to feed addition, centrifuged at 14,500 rpm for 5 min, and stored at −20 °C for subsequent analysis. For low-yield cultures, glucose levels were determined using GlucoSure AutoCode test strips along with a GlucoSure AutoCode blood glucose meter. Lactate concentrations in culture supernatants from low yield cultures (clone 1: days 0, 4, 5, 8, 9 and 12; clone 2: days 0, 3, 4, 5, 6 and 7) were quantified using the L-Lactic Acid Assay Kit (Megazyme) according to the manufacturer's protocol. Samples were diluted and analyzed in triplicate (technical replicates).

For high-yield cultures, glucose, lactate, glutamine, and glutamate levels were measured using a YSI 2900 model biochemistry analyzer (YSI Life Sciences). Ammonium and potassium concentrations were determined with a 2974 ISE Ammonium Electrode and a 2975 ISE Potassium Electrode (YSI Life Sciences) according to the manufacturer's instructions.

### 2.4 Estimation of glucose uptake and lactate production rates

To calculate the cumulative amount of glucose (consumed) and lactate (produced) from the experimental data, equation (1) was used (Tsopanoglou and Jimenez del Val, 2021):

$$\frac{dV{\left[C\right]}_{i}}{dt}=Q_{feed}{\left[C\right]}_{i,feed}-q_{i}\left[X_{v}\right]V$$

$$V{\left[C\right]}_{i}-\int_{n_{0}}^{n}\left(Q_{feed}{\left[C\right]}_{i,feed}\right)dt=-q_{i}\int_{n_{0}}^{n}\left(\left[X_{v}\right]V\right)dt$$

Defining cumulative nutrient consumed up to time *n*, ${CC}_{i,n}$, as:

(1) $${CC}_{i,n}=V{\left[C\right]}_{i}-\int_{n_{0}}^{n}\left(Q_{feed}{\left[C\right]}_{i,feed}\right)dt$$

We obtain a relationship to estimate the specific uptake rate of nutrient *i* over interval *n* ($q_{i,n}$) as a function of the integral of viable cells over the same time interval (${IVC}_{n}$):

(2) $${CC}_{i,n}=-q_{i,n}{IVC}_{n}$$

Where the trapezoidal rule is used to calculate IVC between culture time *t*_*n-1*_ and *t*_*n*_:

(3) $${IVC}_{n}=\sum_{j=1}^{n}\frac{1}{2}\times \left(V_{n}{\left[X_{v}\right]}_{n}+{V_{n-1}[X_{v}]}_{n-1}\right)\times \left(t_{n}-t_{n-1}\right)$$

To estimate the glucose uptake rate and lactate production rate in the different phases of the culture for both clones, linear regression was applied to data for cumulative glucose and lactate consumed against IVC. The slope obtained from the regression indicates the cell-specific glucose uptake rate and lactate production rate, respectively. To estimate the yield coefficient of lactate on glucose, linear regression was performed on data for ${CC}_{Lac}$ and ${CC}_{Glc}$, considering the relationship shown in equation (4).

(4) YLac/Glc=qLacqGlc=CCLac,nIVCnCCGlc,nIVCn=CCLac,nCCGlc,n

The cell-specific growth rate up to time n ($\mu_{g,n}$) and associated doubling time ($t_{d,n}$) were calculated with a similar framework, using equations (5) and (6). Exponential cell-specific growth rate and doubling times were computed for the first 96 h of culture.

$$\frac{dV\left[X_{v}\right]}{dt}=\mu_{g}\left[X_{v}\right]V$$

$$\int_{n_{0}}^{n}dV\left[X_{v}\right]=\mu_{g}\int_{n_{0}}^{n}[X_{v}]Vdt$$

(5) $$V_{n}X_{v,n}=\mu_{g,n}{IVC}_{n}$$

(6) $$t_{d,n}=\frac{\ln\left(2\right)}{\mu_{g,n}}$$

### 2.5 mAb measurement

The concentration of recombinant human IgG in the culture supernatants was measured using a Human IgG ELISA Antibody Pair Kit (STEMCELL Technologies) according to the manufacturer's instructions, or through indirect ELISA as described below.

For indirect ELISA, a high-protein-binding 96-well plate (Nunc Maxisorp™ 96-well ELISA plates, Thermo Fisher Scientific) was coated with 100 μL per well of 2 μg/mL protein A (GenScript) in PBS buffer. After overnight incubation at 4°C, the wells were washed twice with PBS and blocked with a blocking buffer (PBS containing 0.05% Tween 20 and 0.1% bovine serum albumin [BSA]) for 1 h at room temperature. Human IgG standards (STEMCELL Technologies) ranging from 3.2 to 316 ng/mL samples were prepared by diluting them in the blocking buffer. The wells were then washed five times with PBST (PBS containing 0.05% Tween 20). Subsequently, 100 μL of each standard, background control (blocking buffer), and diluted sample were added to the wells and incubated at room temperature for 2 h. The unbound antibodies were removed by washing five times with PBST. Subsequently, 100 μL of diluted Peroxidase AffiniPure F(ab′)2 fragment goat anti-human IgG (H + L) HRP (Jackson ImmunoResearch Laboratories) at a 1:5000 dilution in blocking buffer was added to each well. After incubation for 1 h at room temperature, the wells were washed five times with PBST. The bound antibodies were detected by adding 100 μL of ABTS substrate (ABTS® *BioChemica*, PanReac AppliChem) to each well. After 30 min of incubation at room temperature, the absorbance at 405 nm was measured using a microplate reader (Varioskan LUX, Thermo Scientific) with a correction wavelength of 650 nm.

### 2.6 mAb purification

Recombinant adalimumab in the culture supernatant from the final day of fed-batch culture was centrifuged to remove cells and debris and concentrated using Amicon Ultra centrifugal filters. The antibody was then purified using a NAb Protein A/G spin purification kit (Thermo Fisher Scientific) following the manufacturer's protocol, further concentrated, and stored at −20 °C for subsequent product quality analysis. The procedure followed our previously published method (Pranomphon et al., 2025).

### 2.7 Sample preparation for N-glycan profile analysis

SDS-PAGE analysis and sample preparation for glycan analysis were performed as previously described (Pranomphon et al., 2025). Briefly, purified adalimumab samples were denatured in SDS sample buffer containing 2-mercaptoethanol and heated at 100 °C for 3 min. Approximately 5 μg of protein was loaded onto 10% SDS-PAGE gels and electrophoresed at 200 V for ∼35 min. Gels were stained with Coomassie blue, destained, and rinsed. Heavy-chain bands were cut from the gel and submitted to the BOKU Mass Spectrometry Core Facility (Vienna, Austria) for glycan analysis. The originator Humira product was analyzed in parallel for comparison.

### 2.8 Glycopeptide analysis

Glycopeptide analysis was carried out at the BOKU Core Facility Mass Spectrometry (Vienna, Austria) as previously described (Pranomphon et al., 2025). Briefly, 10–30 μg of protein was subjected to in-solution digestion. Samples were reduced with dithiothreitol and alkylated with iodoacetamide, followed by protein precipitation using ice-cold acetone. The protein pellets were re-dissolved in ammonium bicarbonate buffer and digested overnight with sequencing-grade trypsin at 37 °C. The resulting peptides were separated on a nanoEase C18 column using a 1–40% acetonitrile gradient and analyzed on an Orbitrap Exploris 480 mass spectrometer operated in positive DDA mode. Glycopeptides containing the EEQYNSTYR motif were identified and quantified based on deconvoluted peak intensities using FreeStyle software, as described in our previous study.

### 2.9 Galactosylation and GlcNAc indices

The galactosylation index (GI) reflects the relative abundance of galactose-containing mAb Fc glycoforms and was computed using Equation (7), where $\nu_{k}$ is the number of galactose residues in glycan species k and $x_{k}$ is the relative abundance of glycan species k, as quantified with glycopeptide analysis (Radhakrishnan et al., 2017). Assuming a maximum of two galactose residues per Fc glycan, GI is constrained to values between 0 and 1.

(7) $$GI=\left(\frac{1}{2}\right)\sum_{k=1}^{\#\;Glyc.}\nu_{k}\times x_{k}$$

The GlcNAc index (GNI) similarly quantifies the relative abundance GlcNAc residues in mAb Fc glycoforms and was computed using Equation (8), where $\nu_{l}$ is the number of N-acetylglucosamine residues in glycan species l and $x_{l}$ is the relative abundance of glycan species l, as quantified with glycoprofiling. Assuming a maximum of four GlcNAc residues per Fc glycan (two in the chitobiose core and one on each antenna), the GNI will also be constrained to values between 0 and 1.

(8) $$GNI=\left(\frac{1}{4}\right)\sum_{l=1}^{\#\;Glyc.}\nu_{l}\times x_{l}$$

### 2.10 Computing nucleotide sugar donor (NSD) consumption for cellular and mAb glycosylation

The stoichiometric coefficients of NSD demand towards cellular glycosylation were obtained using equation (9), as derived from Jimenez del Val et al., 2016b:

(9) $$\nu_{{NSD}_{k}}^{Cell\;Nglyc}=\left(\frac{M_{cell}Z_{prot}}{\bar{{MW}_{HCP}}}\right)\left(S_{Nglyc}^{HCP}f_{{NSD}_{k}}^{Nglyc,HCP}\right)$$

In equation (9), $M_{cell}$ is the average weight of CHO cells (Carinhas et al., 2013), $Z_{prot}$ is CHO cell protein content %w/w (Szeliova et al., 2020), $\bar{{MW}_{HCP}}$ is the weighted average molecular weight of CHO HCPs (Jimenez del Val et al., 2016b), $S_{Nglyc}^{HCP}$ is the weighted average number of N-linked glycosites across the CHO proteome, and $f_{{NSD}_{k}}^{Nglyc,HCP}$ is the weighted average monosaccharide composition of CHO HCP N-glycans. $f_{{NSD}_{k}}^{Nglyc,HCP}$ was obtained by multiplying the relative abundance of each N-linked glycan species reported for CHO HCPs (Donini et al., 2025) by the frequency with which each monosaccharide appears in each glycan structure ($n_{{NSD}_{k,i}}$) using equation (10), below:

(10) $$f_{{NSD}_{k}}^{Nglyc,HCP}=\sum_{i=1}^{NGlycans}n_{{NSD}_{k,i}}x_{Nglyc,i}^{HCP}$$

The stoichiometric coefficients for NSD demand towards mAb glycosylation were calculated using equation (11), assuming that each mAb contains two N-linked glycosites in its Fc domain and thus $S_{Nglyc}^{mAb}$ = 2.

(11) $$\nu_{{NSD}_{k}}^{Nglyc,mAb}=\left(\frac{1}{{MW}_{mAb}}\right)S_{Nglyc}^{mAb}f_{{NSD}_{k}}^{Nglyc,mAb}$$

The weighted average monosaccharide composition of mAb glycans ($f_{{NSD}_{k}}^{Nglyc,mAb})$ was obtained from equation (12) using the stoichiometric coefficient of monosaccharide k ($n_{{NSD}_{k,j}}$) in glycan j and the experimentally measured relative abundance of each N-linked glycan species ($x_{Nglyc,j}^{mAb}$):

(12) $$f_{{NSD}_{k}}^{Nglyc,mAb}=\sum_{j=1}^{mAbGlycans}n_{{NSD}_{k,j}}x_{Nglyc,j}^{mAb}$$

To estimate NSD demand towards HCP and mAb glycosylation, the calculated stoichiometric coefficients were multiplied by the specific growth rate ($\mu_{g})$ and specific productivity ($Q_{p})$ for each clone in the low-yield and high-yield culture conditions, as shown in equations (13) and (14).

(13) $$D_{{NSD}_{k}}^{Nglyc,HCP}=\nu_{{NSD}_{k}}^{Cell\;Nglyc}\mu_{g}$$

(14) $$D_{{NSD}_{k}}^{Nglyc,mAb}=\nu_{{NSD}_{k}}^{Nglyc,mAb}Q_{p}$$

### 2.11 Statistical analysis

Statistical analyses were performed using GraphPad Prism version 11.0.1 for Windows, GraphPad Software, San Diego, CA, USA, www.graphpad.com.

## 3 Results and discussion

### 3.1 Cell growth, metabolism, and antibody production in high- and low-yield shake-flask cultures

Clones 1 (DHFR-based) and 2 (GS-based) were cultured under low- and high-yield fed-batch shake-flask conditions, with high-yield conditions incorporating increased agitation rate (150 rpm vs. 130 rpm), increased GlutaMAX supplementation for clone 1, and increased anti-clumping agent for clone 2. These modifications resulted in substantially enhanced cell growth and volumetric productivity in both cell lines, accompanied by changes in central carbon metabolism and mAb glycosylation patterns.

**Fig. 1:**
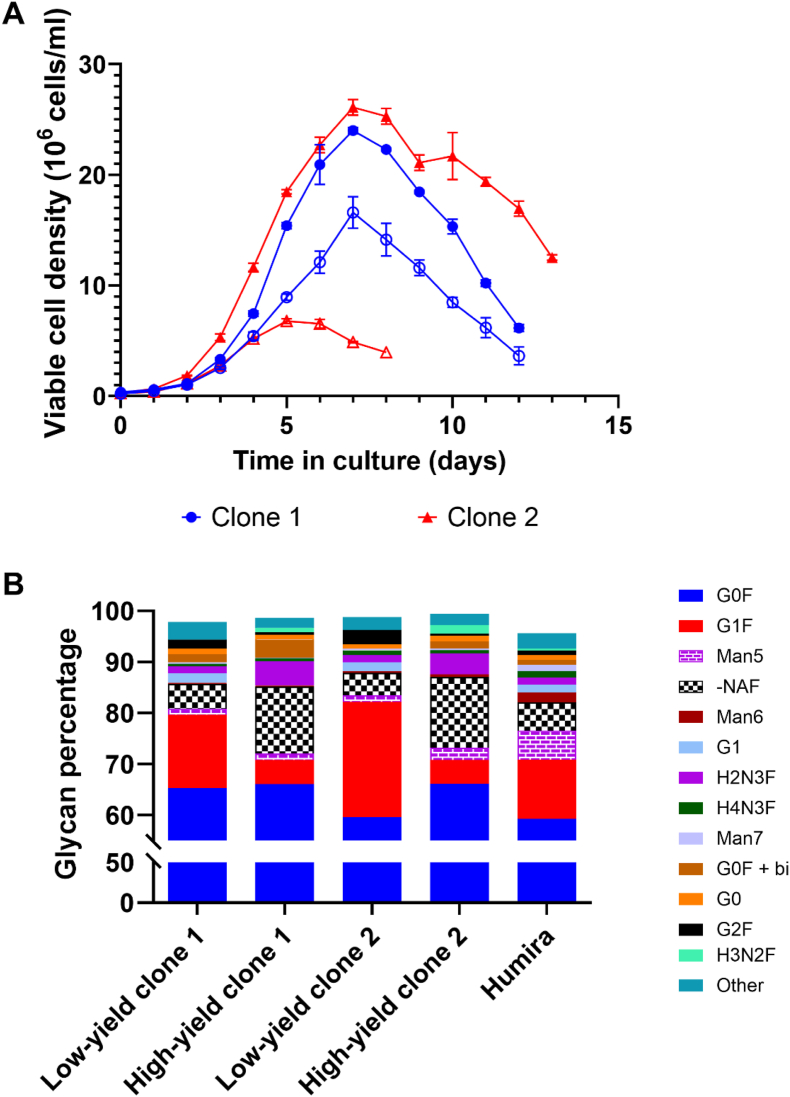
(A) Viable cell densities (VCD) of clones 1 and 2 cultured in low-yield (open symbols) and high-yield (filled symbols) culture conditions. Error bars indicate standard deviation (n = 2). (B) Glycosylation profiles of adalimumab produced by clones 1 and 2 under low-yield and high-yield culture conditions compared with glycan profiles of originator Humira.

Under high-yield conditions, clone 1 reached a 1.4-fold higher peak viable cell density (VCD) and a 1.6-fold higher integral of viable cells (IVC) compared with low-yield cultures (Fig. 1A, Table 1). This increase translated to a final antibody titer of 102.66 mg/L, representing a 1.3-fold improvement. Clone 2 exhibited a stronger response, with a 3.9-fold higher peak VCD and 7.4-fold greater IVC under high-yield conditions, resulting in a fourfold increase in product titer (Fig. 1A, Table 1). Although specific productivity was reduced for both clones in high-yield cultures, the reduction was offset by the higher IVC, yielding higher final titers (Table 1).

**Table 1:** Comparison of growth and productivity of clones 1 and 2 under low-yield and high-yield culture conditions.

Culture condition	Peak IVC (10^6^ cells×day)		Qp (pg/cell/day)		Doubling Time (hrs)		Titer (mg/L)	
	Clone 1	Clone 2	Clone 1	Clone 2	Clone 1	Clone 2	Clone 1	Clone 2
Low yield	2,508 ± 179	793 ± 90	0.935± 0.033	0.676 ± 0.010	21.85 ± 0.47	23.14 ± 1.34	80.7 ± 2.3	19.2 ± 2.6
High yield	4,115 ±110	5,641 ± 149	0.778 ± 0.059	0.415± 0.013	21.30 ± 0.80	19.99 ± 0.79	102.7 ± 6.1	76.9 ± 3.3

Average doubling times during exponential growth, computed from $\mu_{g}$ (Supplementary Fig. S1) with equation (6), decreased under high-yield conditions for both clones, from 21.9 ± 0.5 h to 21.3 ± 0.8 h for clone 1 and from 23.1 ± 1.3 h to 20.0 ± 0.8 h for clone 2 (Table 1). Notably, high-yield clone 2 cultures were sustained for 13 days compared with 8 days in low-yield cultures, whereas culture duration for clone 1 was not extended (Fig. 1A).

Glucose and lactate concentrations were monitored throughout cultivation in both low-yield (Supplementary Fig. S2A–B) and high-yield cultures (Supplementary Fig. S3A–B). Glucose concentrations were maintained at ∼6 g/L using an alternating glucose/EfficientFeed C+ feeding strategy, and no cultures experienced glucose limitation (Supplementary Fig. S2A and S3A). Final glucose concentrations in high-yield cultures decreased to below ∼4 g/L, compared with 4–5 g/L in low-yield cultures, consistent with increased cellular growth.

In both low- and high-yield cultures, lactate was initially produced and subsequently consumed toward the end of cultivation, indicating a metabolic shift from lactate production to lactate uptake (Supplementary Fig. S2B and S3B). Phase-specific linear regression analyses of specific glucose uptake rates and lactate production rates are shown in Supplementary Fig. S4 and S5 and Table 2. High-yield cultures exhibited slightly reduced specific glucose uptake and lactate production rates but a significant decrease in the yield coefficient of lactate from glucose relative to low-yield cultures (Table 2, Supplementary Fig. S6). This reduction indicates that a greater fraction of glucose was directed towards oxidative phosphorylation rather than aerobic glycolysis under high-yield conditions.

**Table 2:** Comparison of specific glucose uptake rate, specific lactate production rate and yield of lactate from glucose for clones 1 and 2 under low-yield and high-yield culture conditions.

Culture condition	Glucose uptake rate (mg/day/10^6^ cells)			Lactate production rate (mg/day/10^6^ cells)			Yield of lactate from glucose (mmol/mmol)		
	Exponential	Stationary	Death	Exponential	Stationary	Death	Exponential	Stationary	Death
Low yield clone 1	0.534 ± 0.035	0.203 ± 0.003	0.345 ± 0.012	0.237 ± 0.006	−0.023 ± 0.001	0.009 ± 0.000	0.95 ± 0.01	−0.22 ± 0.01	0.053 ± 0.007
High yield clone 1	0.561 ± 0.077	0.163 ± 0.048	0.252 ± 0.033	0.187 ± 0.012	−0.011 ± 0.001	0.002 ± 0.000	0.67 ± 0.02	−0.15 ± 0.01	0.009 ± 0.002
Low yield clone 2	0.584 ± 0.043	0.294 ± 0.014	0.287 ± 0.015	0.549 ± 0.03	−0.008 ± 0.004	−0.061 ± 0.004	2.00 ± 0.06	−0.08 ± 0.02	−0.38 ± 0.04
High yield clone 2	0.395 ± 0.11	0.121 ± 0.054	0.164 ± 0.003	0.304 ± 0.027	−0.011 ± 0.001	0.012 ± 0.004	0.78 ± 0.07	−0.21 ± 0.02	0.11 ± 0.05

These metabolic changes are consistent with enhanced oxygen availability resulting from increased agitation and, in clone 2, reduced cell clumping due to anti-clumping agent addition. Improved oxygen mass transfer would be expected to promote oxidative metabolism, increase ATP yield per mole of glucose, reduce lactate secretion, and support higher growth rates and IVC, as previously reported (Yin et al., 2025; Zagari et al., 2013).

Glutamine, glutamate, ammonium, and potassium concentrations were monitored in high-yield cultures (Supplementary Fig. S3C–F). In high-yield clone 1 cultures supplemented with GlutaMAX, free glutamine concentrations remained low during the first day (<0.2 mM) and increased to approximately 1 mM by day 3, coincident with rapid cell growth and increased aminopeptidase activity (Supplementary Fig. S3C). To avoid glutamine depletion during exponential growth, additional GlutaMAX was added on day 5. Glutamine was subsequently depleted at a high rate during exponential growth and increased again late in culture (days 10–12), likely due to cell death and amino acid release.

In contrast, clone 2, a GS-based cell line grown without glutamine supplementation, maintained relatively stable glutamine levels throughout culture, followed by a gradual increase to approximately 1.3 mM at harvest (Supplementary Fig. S3C). Glutamate concentrations increased steadily in both cultures due to feed composition (Supplementary Fig. S3D), though levels were slightly lower in clone 2, consistent with its utilization for endogenous glutamine synthesis. Ammonium accumulated steadily in both cultures (Supplementary Fig. S3E), with clone 2 exhibiting lower concentrations than clone 1, as expected for GS-based metabolism. Potassium concentrations remained similar across cultures (Supplementary Fig. S3F).

### 3.2 Glycan analysis

**Fig. 2:**
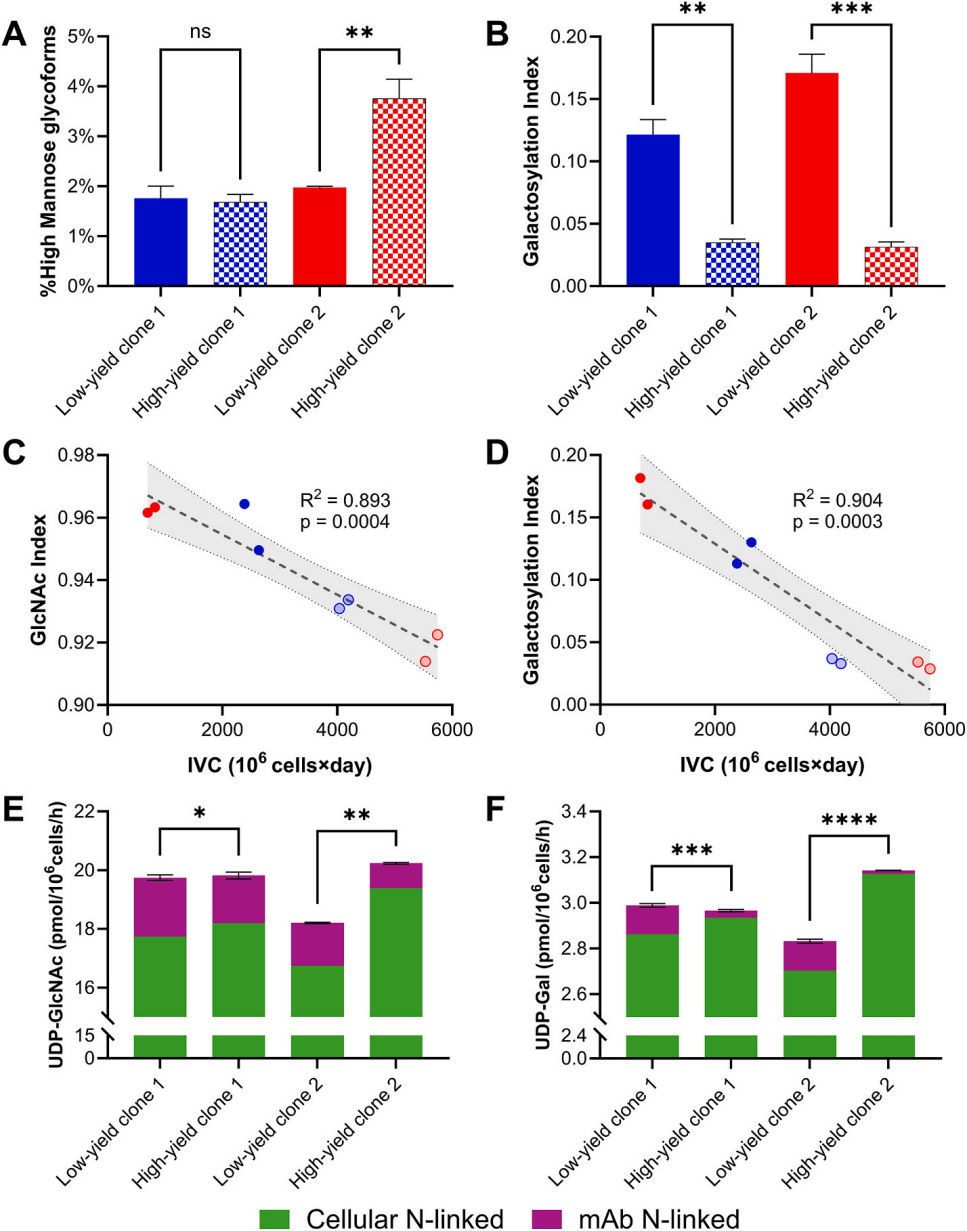
(A) Total relative abundance of high mannose (HM) glycans of clones 1 and 2 cultured in low-yield (solid bars) and high-yield (patterned bars) culture conditions. Error bars indicate standard deviation (n = 2). (B) Galactosylation index computed from measured glycosylation profiles of adalimumab produced by clones 1 and 2 under low- and high-yield culture conditions (n = 2). (C) Correlation between endpoint IVC and mAb GlcNAc Index. (D) Correlation between endpoint IVC and Galactosylation Index. (C) and (D) present all individual replicates for low-yield (dark fill) and high-yield (light fill) cultures of clones 1 (blue) and 2 (red). (E) UDP-GlcNAc flux towards cellular and mAb glycosylation for low-yield and high-yield cultures. (F) UDP-Gal flux towards cellular and mAb glycosylation for low-yield and high-yield cultures. Unpaired t-tests were performed to compare the mean values between low-yield and high-yield cultures (A and B). For (E) and (F) t-tests compare differences in the fluxes towards mAb N-linked glycosylation. ns denotes no statistically significant difference, * corresponds to p < 0.05, ** to p < 0.01, *** to p < 0.001, and **** to p < 0.0001. Linear regression was performed for (C) and (D). The shaded area corresponds to the 95% confidence interval of the regression, and the R^2^ and p-values correspond to the coefficient of determination and statistical significance of the linear regression slope, respectively.

N-linked glycan profiles of adalimumab produced by both clones under low- and high-yield conditions were analyzed and compared with the originator Humira (Fig. 1B, Table 3; nomenclature per (Peng et al., 2018), Supplementary Scheme S1). Transitioning to high-yield conditions resulted in consistent shifts toward less processed glycoforms in both clones, as reflected by increased accumulation of -NAF (Fig. 1B) and high mannose glycans (Fig. 2A, Table 3) as well as reductions in galactosylated glycoforms.

**Table 3:** Variation in the most significant glycans in adalimumab produced by clones 1 and 2 in low-yield and high-yield cultures.

GlycanCulture condition	G0F		G1F		Man5		-NAF		H2N3F	
	%	% change from low-yield culture	%	% change from low-yield culture	%	% change from low-yield culture	%	% change from low-yield culture	%	% change from low-yield culture
Low yield clone 1	65	N/A	14	N/A	1	N/A	5	N/A	1	N/A
High yield clone 1	66	0.8	5	−9.6	1	N/A	13	8.4	5	3.5
Low yield clone 2	60	N/A	23	N/A	1	N/A	4.4	N/A	1	N/A
High-yield clone 2	66	6.5	5	−17.9	2	1.1	14	9.4	4	2.7

Monogalactosylated G1F decreased significantly by 9.6% in clone 1 and 17.9% in clone 2, while bi-galactosylated G2F declined modestly. A reduction in overall galactosylation can be seen in Fig. 2B, where statistically significant differences are observed in the GI obtained for low- and high-producing cultures across both clones. G0F increased modestly in clone 1 and more notably in clone 2 under high-yield conditions, while Man5 remained unchanged in clone 1 but increased slightly and significantly in clone 2 (Table 3). Non-glycosylated species decreased slightly (<1%), and G1 species were not detected in either high-yield culture. G0 levels decreased slightly in high-yield clone 1 cultures but increased modestly in clone 2 cultures.

Comparison with Humira revealed clone- and condition-dependent differences. In low-yield cultures, clone 1 exhibited higher G0F levels than Humira, whereas clone 2 was comparable (Fig. 1B). In high-yield cultures, both clones exceeded Humira in G0F content while exhibiting lower G1F and higher –NAF levels. High-mannose species (Man5–Man7) remained at low levels (<1%) and were lower than in Humira, except for the slight Man5 increase observed in high-yield clone 2 cultures. G0 and G2F levels in high-yield cultures were comparable to those of Humira.

### 3.3 Impact of growth and productivity on nucleotide sugar donor availability and mAb glycosylation patterns

Our experimental results show that increased agitation leads to higher mAb titers. However, the higher titers arise from increased IVC and not due to enhancement of $Q_{p}$ (Table 1). Considerable improvement in glucose metabolism was also observed, with the high-yielding cultures presenting substantially lower yields of lactate on glucose during exponential growth (Table 2). Because a lower $Q_{p}$ potentially translates into longer residence time for glycan processing in the Golgi apparatus (Jimenez del Val et al., 2016a; Madabhushi et al., 2021; Pranomphon et al., 2025), MGAT1 and β1,4-galactosyltransferase availability are less likely to cause the observed increase in high mannose glycans in clone 2 (Fig. 2A) and the reduction in mAb galactosylation (Fig. 2B). Although measurement of glycosylation gene expression would be required to confirm this premise, studies examining the effects of nutrient availability, temperature shift, and Mn^2+^/Gal feeding on CHO cell growth and mAb glycosylation report that MGAT1 and β1,4-galactosyltransferase transcription/expression levels vary modestly, if at all, despite substantial changes in culture conditions (Fan et al., 2015a, 2015b; Pranomphon et al., 2025).

More efficient glucose utilization suggests less constrained UDP-GlcNAc and UDP-Gal biosynthesis; therefore, reduced NSD *supply* is unlikely to account for the observed reductions in mAb galactosylation. While direct measurements of intracellular NSD pools would confirm this, previous work has shown that UDP-GlcNAc and UDP-Gal accumulate within CHO cells when sufficient carbon sources are available (Kochanowski et al., 2008; Kotidis et al., 2019), suggesting that NSD supply is unlikely to be limiting in our high-yield culture conditions.

A strong, negative correlation between glycan processing and IVC is observed across both cell lines and culture conditions (Fig. 2C and D), where the highest IVC values correspond to the lowest GlcNAc and galactosylation indices (GNI and GI, respectively). In contrast, correlations between GI and both $\mu_{g}$ and $Q_{p}$ are not statistically significant (p ≥ 0.07), while GNI correlations with $\mu_{g}$ and $Q_{p}$ are weaker (R^2^ ≤ 0.64), although still statistically significant (p ≤ 0.05) (Supplementary Fig. S7). Together, these findings suggest that increased growth may deplete the metabolic resources required for glycosylation in these cell lines and culture conditions.

We posit that NSD *demand* towards increased cell proliferation drives the observed glycosylation shifts in these cell lines. To investigate this hypothesis, the cell-specific uptake fluxes of UDP-GlcNAc and UDP-Gal towards cellular and mAb N-linked glycosylation were computed for both clones under low- and high-yield conditions using equations (8), (9), (10), (11), (12), (13), (14) (Fig. 2E and F) (Jimenez del Val et al., 2016b). For both clones, the computed rate of UDP-GlcNAc consumption towards cellular N-linked glycosylation was higher in the high-yield culture conditions, caused by the observed decrease in doubling time (increase in specific growth rate). A concomitant decrease in the rate of UDP-GlcNAc consumption towards mAb glycosylation was also observed (Fig. 2E).

The reductions in UDP-Gal consumption rates towards mAb glycosylation when moving from low to high-yield cultures is even more pronounced (Fig. 2F). The reductions in NSD fluxes towards mAb glycosylation are partially due to reduced Qp when shifting from low-to high yield cultures. In particular, the downshift in Qp may underly the reduction in UDP-GlcNAc consumption across both clones (Supplementary Fig. S8). However, the notable accumulation of -NAF (Fig. 1B) suggests that reduced glycan processing may also contribute to the reduced UDP-GlcNAc flux towards mAb. Well over half of the reduction in UDP-Gal flux arises due to shortfalls in mAb glycosylation (Supplementary Figure S8 and Fig. 2B).

Within our NSD supply/demand hypothesis, the shortfalls in UDP-Gal consumption towards mAb glycosylation are more likely to arise from increased NSD demand towards cellular glycosylation. It is this added demand, which is due to considerably enhanced growth (Fig. 1A), that depletes UDP-Gal and, ultimately, limits mAb glycan processing. To possibly compound the demand-side effect of cellular glycosylation, the ammonium concentrations observed in later stages of the high-yield cultures (Supplementary Fig. S3) approximate the 0.2 g/L threshold, where this metabolite is reported to impact glycosylation (Borys et al., 1994; Chen and Harcum, 2006).

## 4 Conclusions

Enhancing yield is crucial for patient access to biosimilars because cost of goods has a disproportionate impact on revenue and, therefore, economic viability. Manipulating the physicochemical environment can increase yield but may also adversely affect mAb quality. Here, we demonstrate that increasing agitation speed in fed-batch shake flask cultures significantly enhances yield, likely through improved oxygen transfer, with a concomitant alteration of glycosylation patterns across the tested CHO cell lines and culture conditions. Our computational analysis suggests that, in the tested conditions, increased NSD demand towards cellular glycosylation is an important contributor to the observed shifts. We acknowledge that the simultaneous variation of multiple culture parameters (agitation speed, GlutaMAX supplementation, and anti-clumping reagent concentration) limits our ability to isolate the contribution of individual factors; however, the consistent negative correlation between IVC and glycan processing across both cell lines and all culture conditions supports the validity of our conclusions. Future work will address these limitations through experimental validation, including: (i) intracellular NSD profiling under the different culture conditions, (ii) qPCR to rule out differential glycogene expression, and (iii) cellular glycoprofiling.

## Declaration of generative AI and AI-assisted technologies in the manuscript preparation process

During the preparation of this work, the author(s) used ChatGPT 5.0 to revise the manuscript to improve clarity and conciseness. The author(s) reviewed and edited the output as needed and take full responsibility for the content of the published article.

## CRediT authorship contribution statement

**Ranya Pranomphon:** Formal analysis, Investigation, Methodology, Writing – original draft. **Sofia Gialamoidou:** Formal analysis, Investigation, Methodology, Writing – original draft. **Montarop Yamabhai:** Conceptualization, Funding acquisition, Project administration, Supervision, Writing – review & editing. **Susan T. Sharfstein:** Conceptualization, Funding acquisition, Project administration, Resources, Supervision, Writing – review & editing. **Ioscani Jiménez del Val:** Conceptualization, Funding acquisition, Project administration, Supervision, Writing – review & editing.

## Declaration of competing interest

The authors declare that there are no conflicts of interest.

## Data Availability

Data will be made available on request.
