# Supplementary material for: Experimental and computational analysis of glycan processing at increased monoclonal antibody productivities
Source: Metab Eng Commun. 2026 Jul 20;23:e00285. doi: 10.1016/j.mec.2026.e00285 (PMC13452297; doi:10.1016/j.mec.2026.e00285)
Supplement: Supplementary file 1 [file mmc1.pdf]

Supplementary Materials

Experimental and Computational Analysis of Glycan Processing at Increased Monoclonal Antibody Productivities

Ranya Pranomphon[1,2,†], Sofia Gialamoidou[3,†], Montarop Yamabhai[2], Susan T. Sharfstein[1], and Ioscani Jiménez del Val[3,*]

1. College of Nanotechnology, Science and Engineering and The RNA Institute, University at Albany, State University of New York, Albany, NY 12203, USA
2. School of Biotechnology, Institute of Agricultural Technology, Suranaree University of Technology, Nakhon Ratchasima, Thailand
3. School of Chemical & Bioprocess Engineering, University College Dublin, D04 V1W8 Dublin, Ireland

* To whom all correspondence should be addressed: ioscani.jimenezdelval@ucd.ie

† These two authors contributed equally to this manuscript.

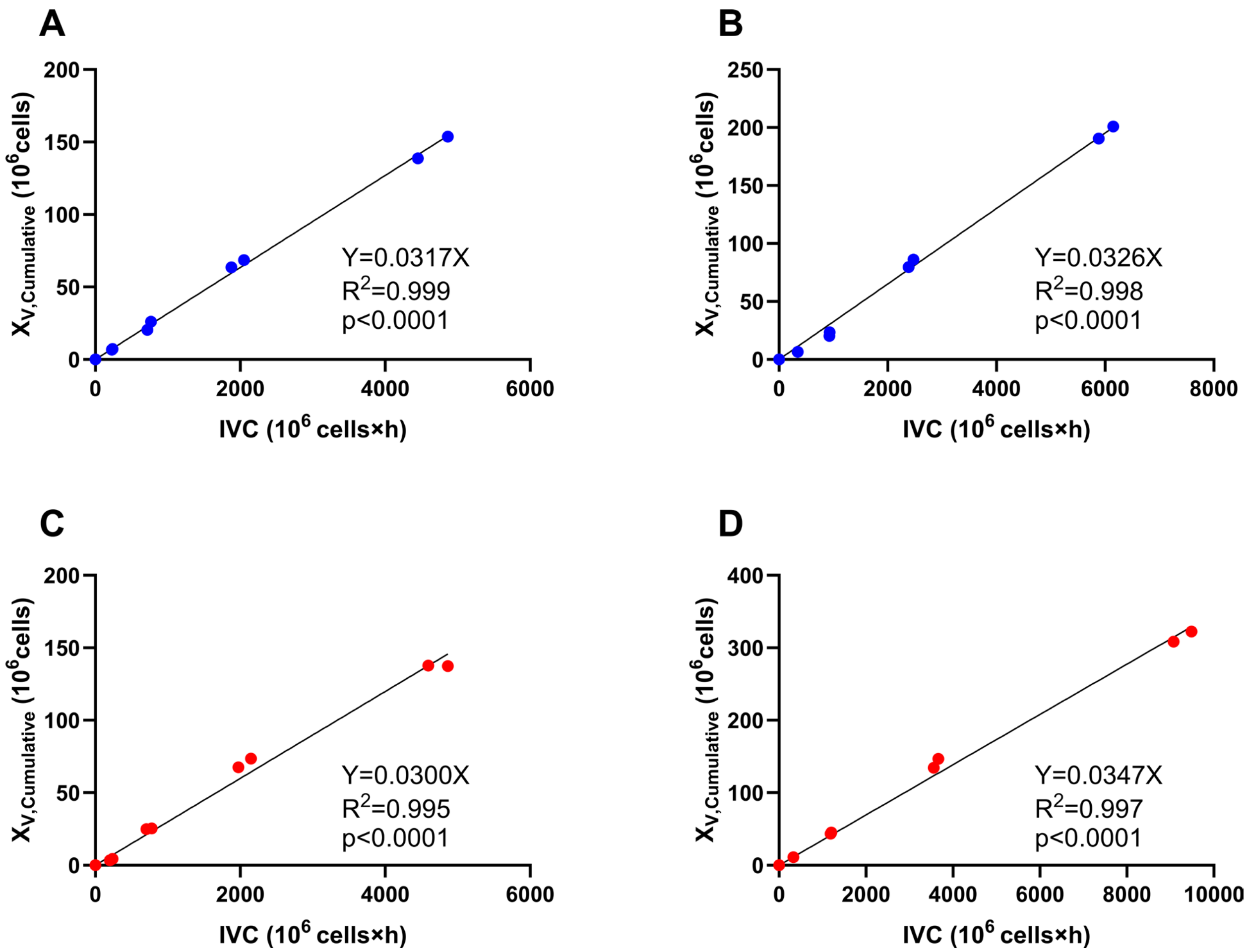

Figure S1: Linear regression plots of cumulative viable cell density ($X_{v,c}$) vs integral of viable cells (IVC). A) Clone 1 in low-yield culture conditions; B) Clone 1 in high-yield culture conditions; C) Clone 2 in low-yield culture conditions; D) Clone 2 in high-yield culture conditions. The data corresponds to the first 96 hours of culture, which corresponds to the exponential growth phase. The obtained slope equates to the maximum cell specific growth rate ($\mu_{g,max}$). Linear regression and statistical analyses were performed on GraphPad Prism version 11.0.1 for Windows, GraphPad Software, San Diego, CA, USA, www.graphpad.com.

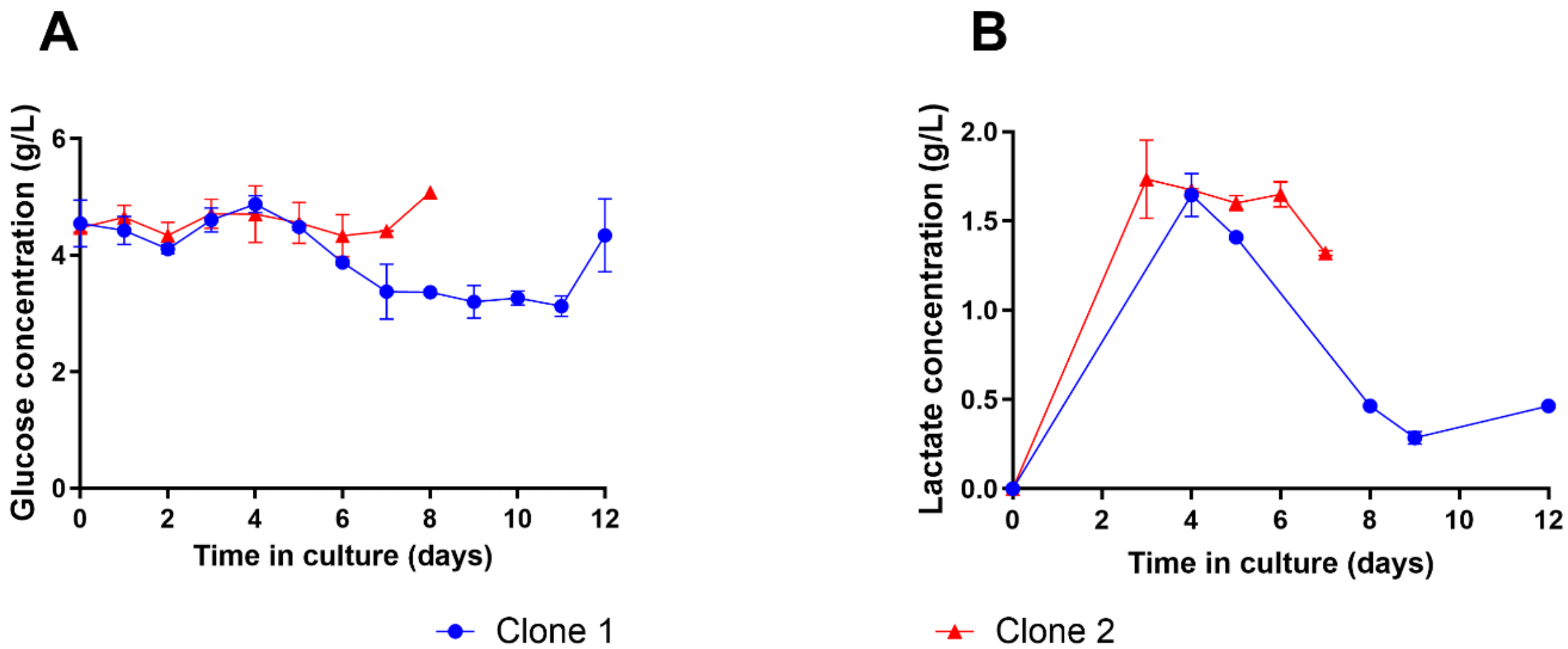

Figure S2: Metabolic behavior of clones 1 and 2 in low-yield cultures: (A) glucose concentration and (B) lactate concentration. Error bars represent the standard deviation of duplicate biological samples.

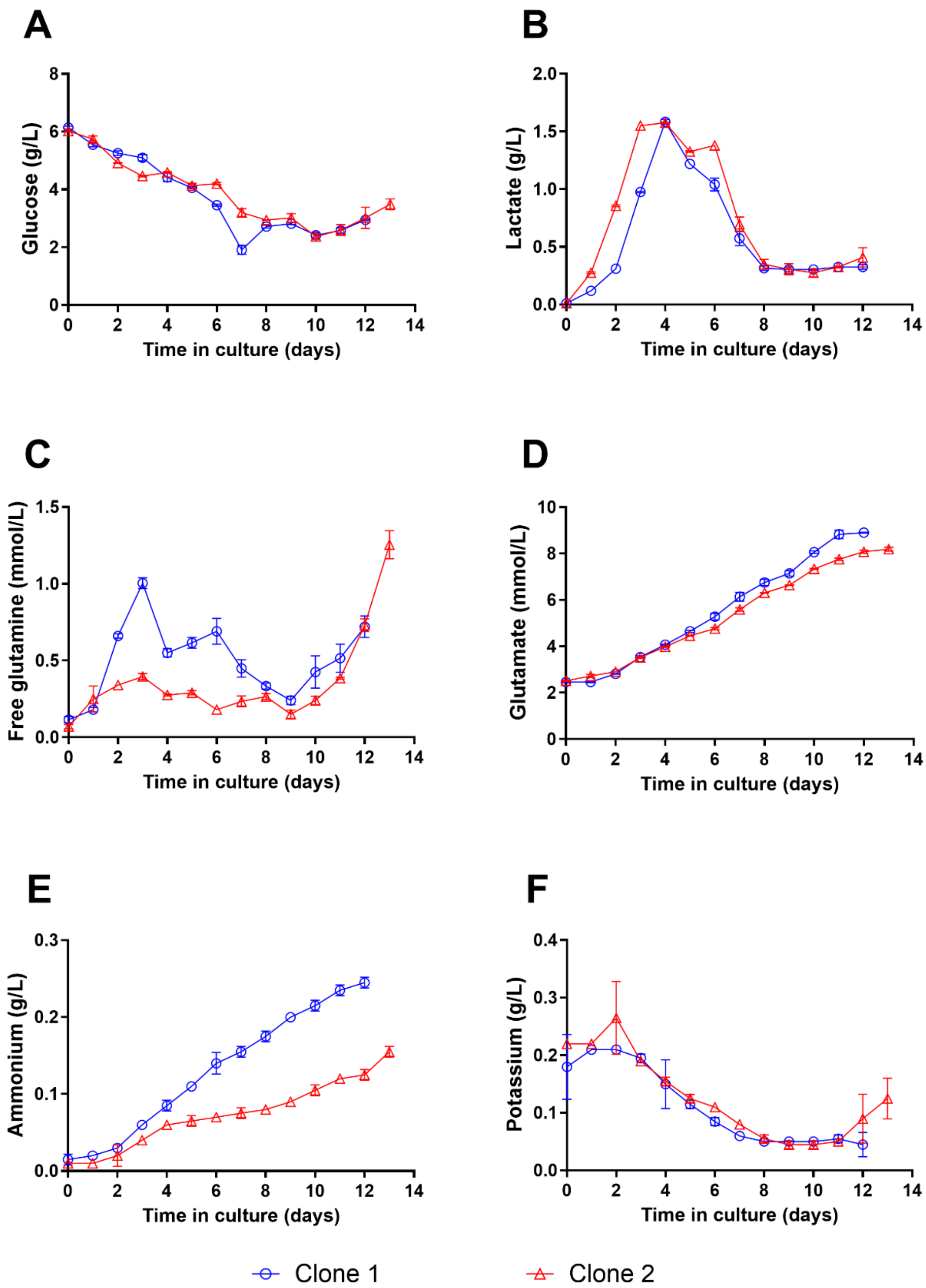

Figure S3: Key metabolites of clones 1 and 2 in high-yield cultures. (A) glucose, (B) lactate, (C) glutamine, (D) glutamate, (E) ammonium, and (F) potassium. Error bars represent the standard deviation of duplicate biological samples.

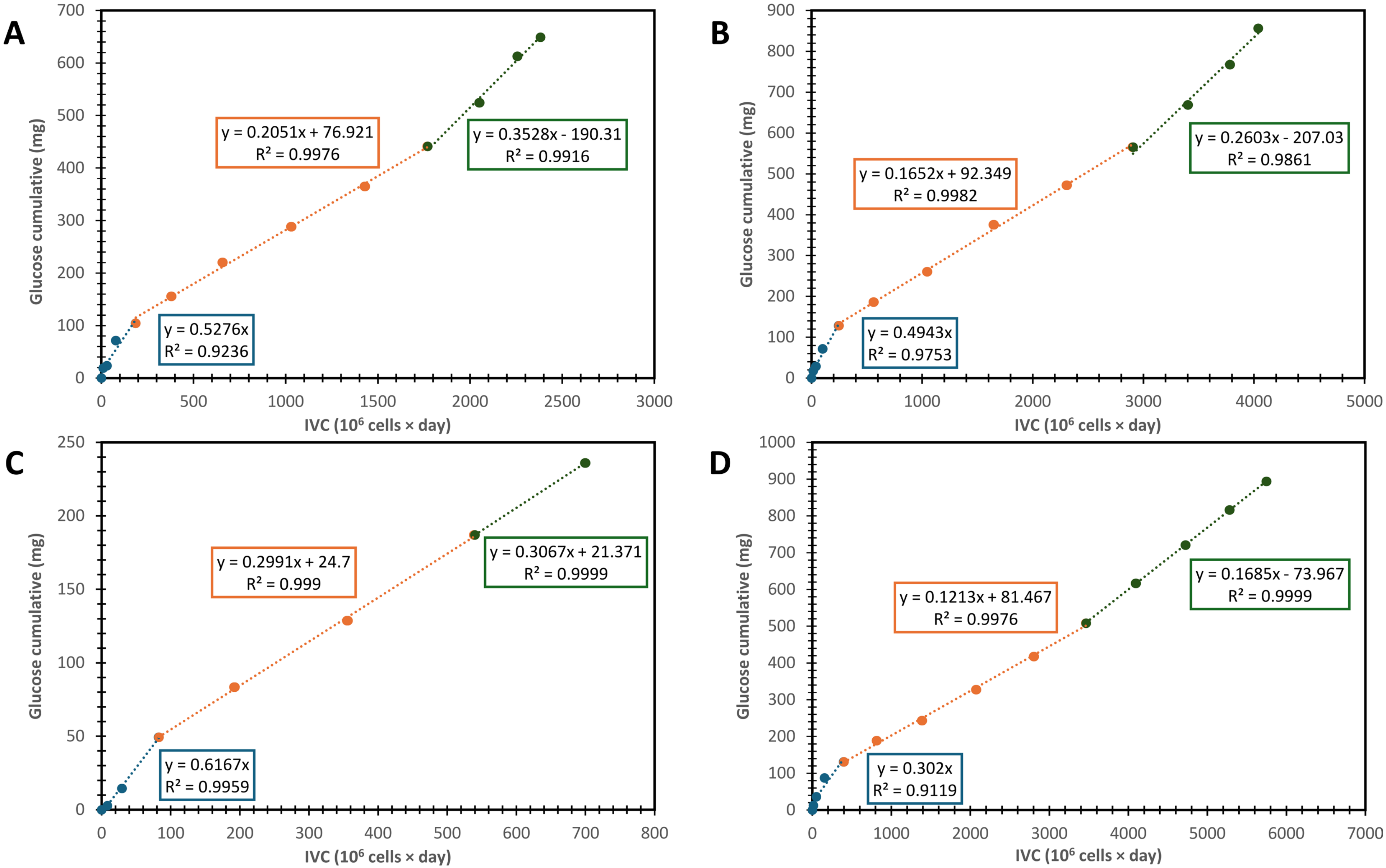

Figure S4: Linear regression plots of cumulative glucose uptake vs IVC for A) Clone 1 in low-yield culture conditions; B) Clone 1 in high-yield culture conditions; C) Clone 2 in low-yield culture conditions; D) Clone 2 in high-yield culture conditions.

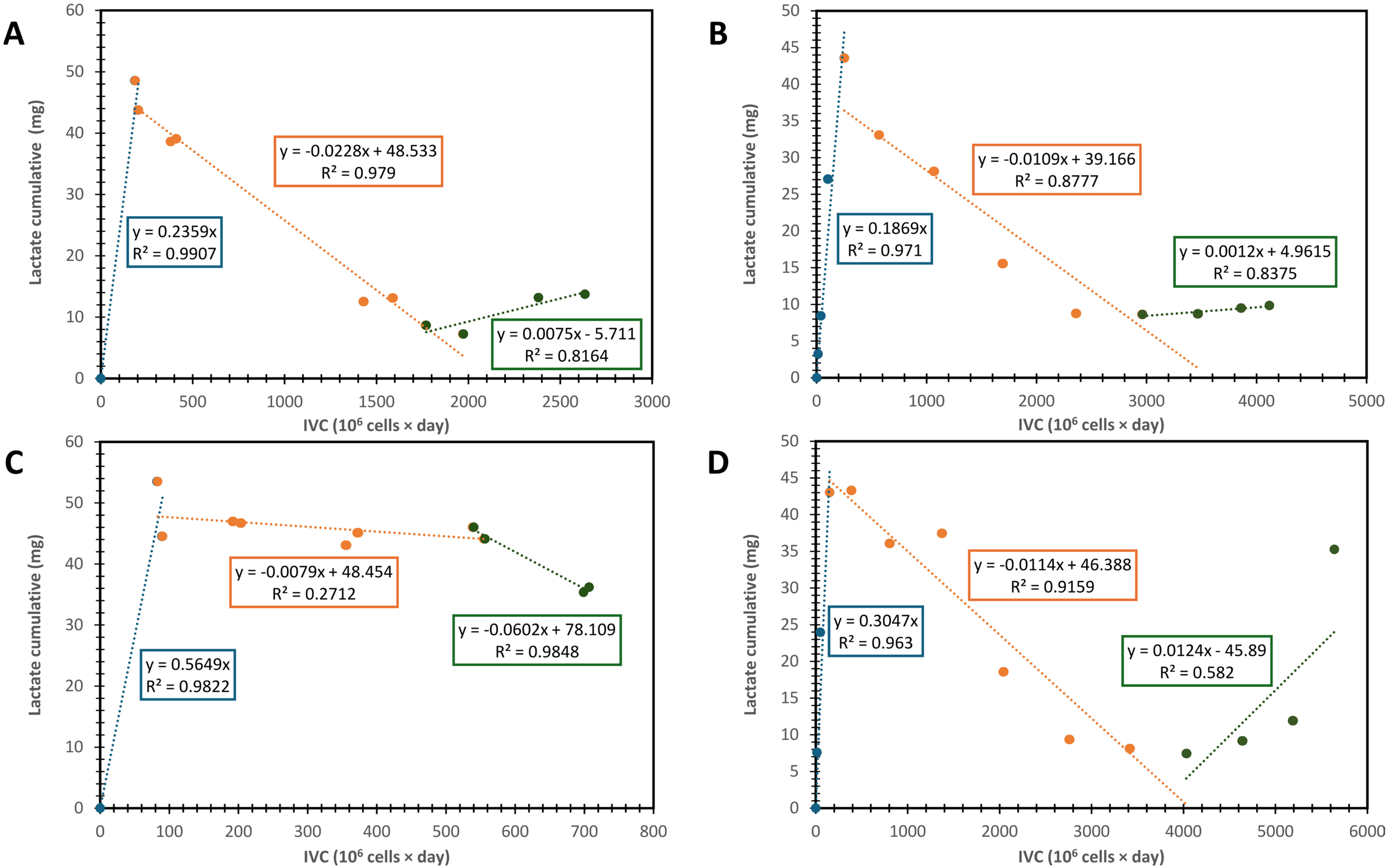

Figure S5: Linear regression plots of cumulative lactate production vs IVC for A) Clone 1 in low-yield culture conditions; B) Clone 1 in high-yield culture conditions; C) Clone 2 in low-yield culture conditions; D) Clone 2 in high-yield culture conditions.

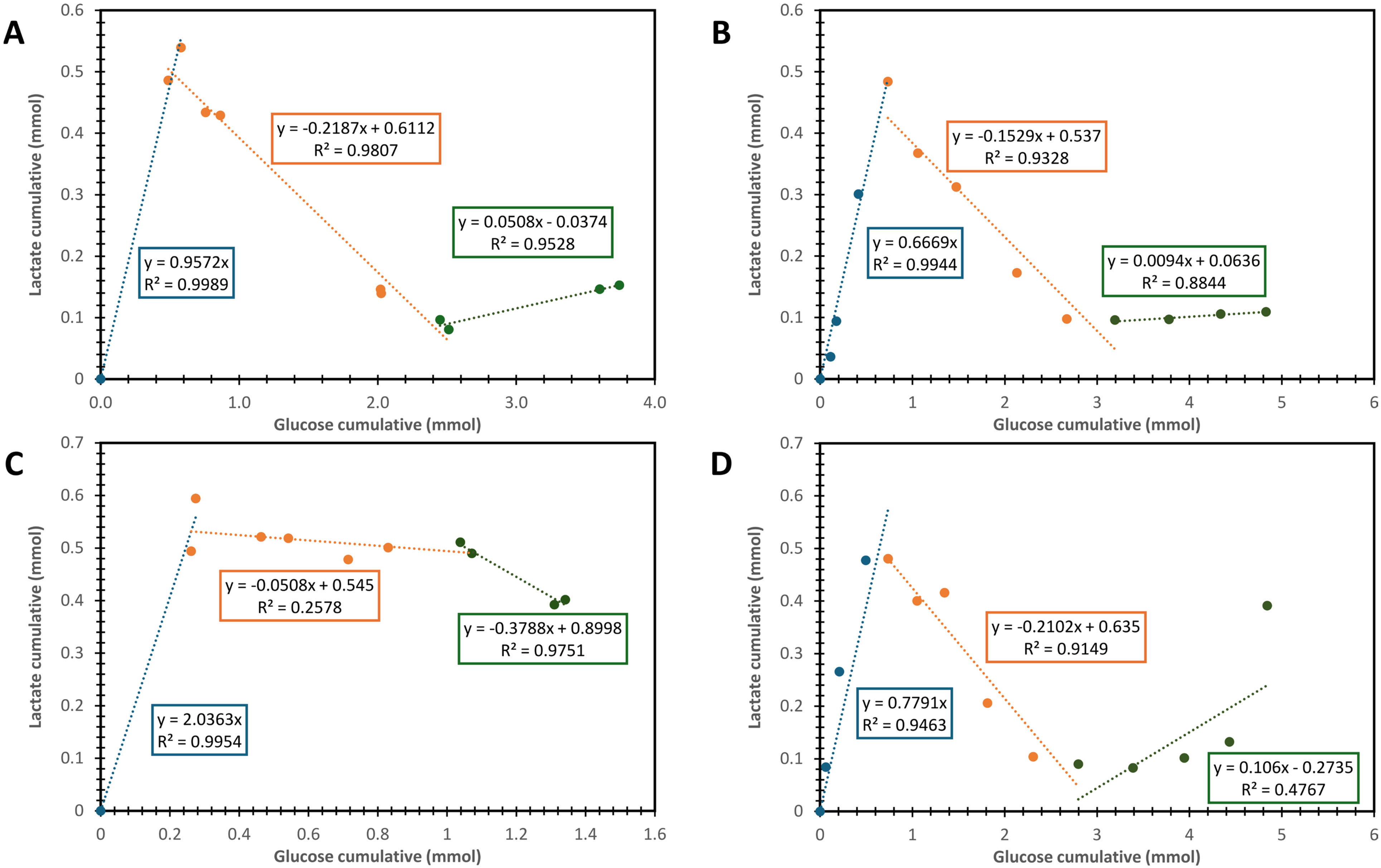

Figure S6: Linear regression plots of cumulative lactate produced vs cumulative glucose consumed for A) Clone 1 in low-yield culture conditions; B) Clone 1 in high-yield culture conditions; C) Clone 2 in low-yield culture conditions; D) Clone 2 in high-yield culture conditions.

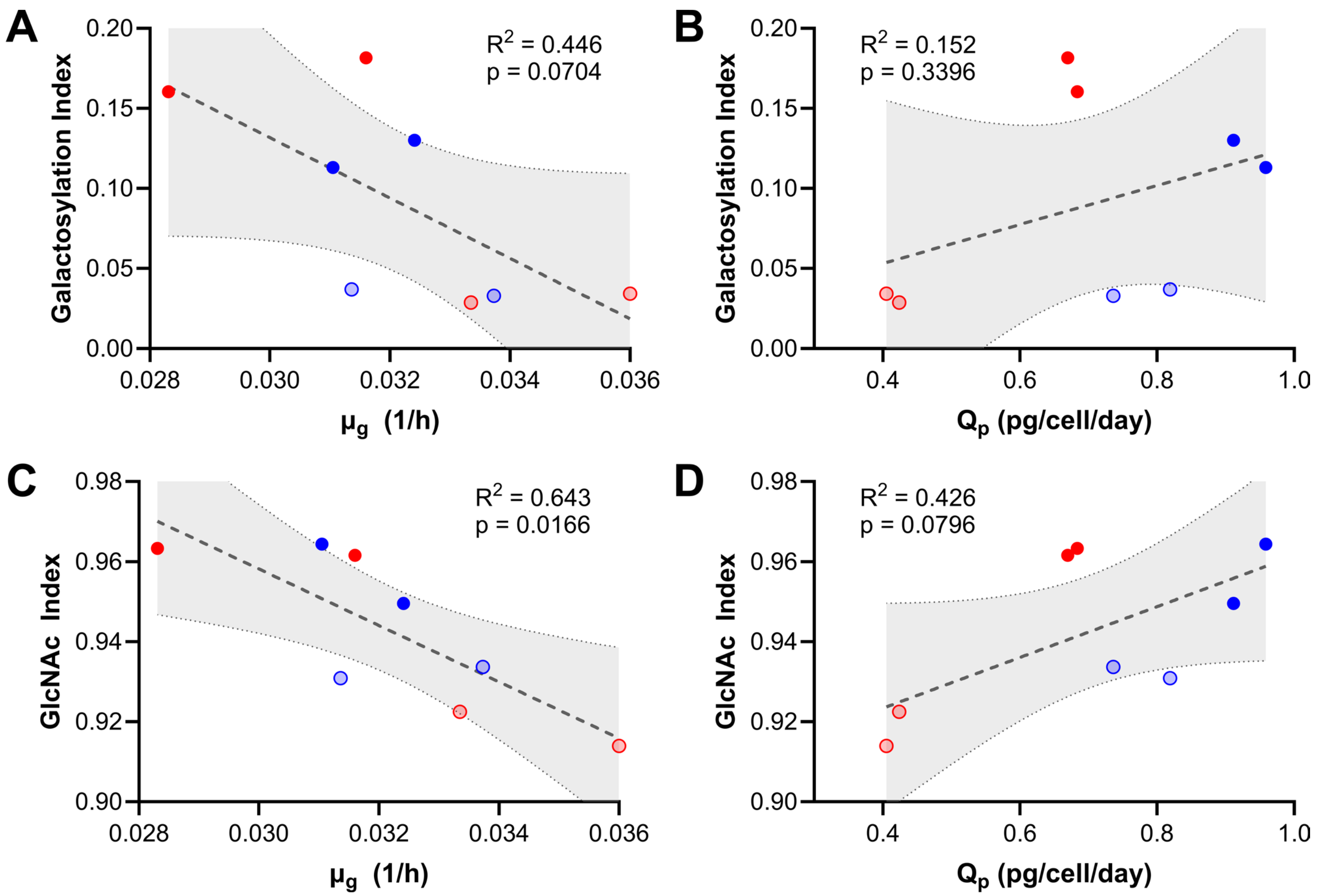

Figure S7: Correlation analysis for GlcNAc Index and Galactosylation Index with respect to specific growth rate (μ_g) and cell-specific productivity (Q_p). The data in each panel spans clones 1 (blue) and 2 (red) cultured under low- (dark fill) and high-producing (light fill) conditions. The shaded area corresponds to the 95% confidence interval of the linear regression. R^2 is the coefficient of determination and the p-value corresponds to the statistical significance of the linear regression slope. Correlation analyses were performed on GraphPad Prism version 11.0.1 for Windows, GraphPad Software, San Diego, CA, USA, www.graphpad.com.

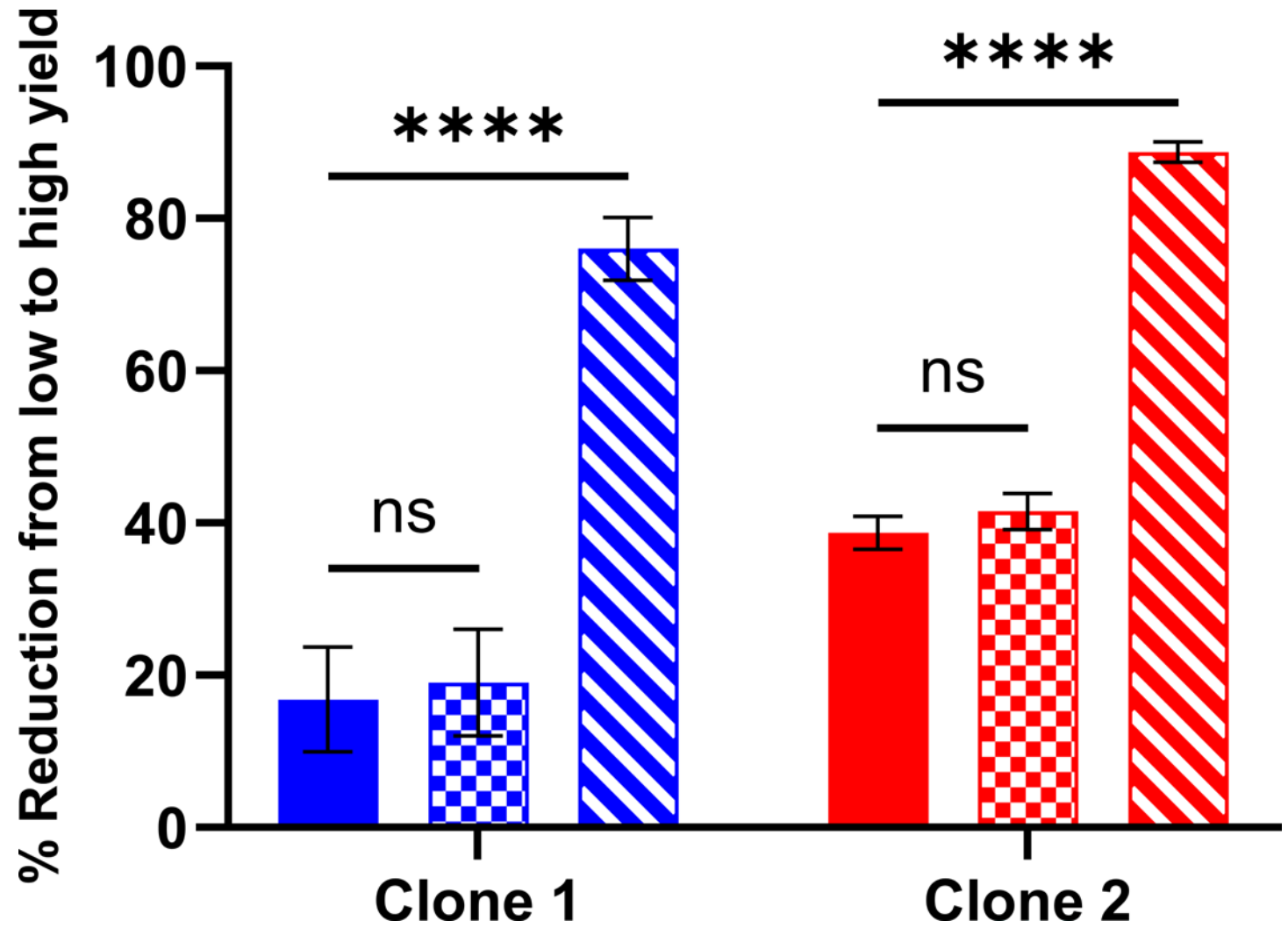

Figure S8: Percent reduction in Qp and NSD fluxes towards mAb glycosylation upon shifting from low to high-yield culture. The solid bars show the percent reduction in Qp, the checkered bars correspond to the reduction in UDP-GlcNAc flux towards mAb glycosylation, and the diagonal patterned bars show the reduction in UDP-Gal flux towards mAb glycosylation. Unpaired t-tests were performed to compare the mean values between Qp and the NSD fluxes. ns denotes no statistically significant difference, * corresponds to $p < 0.05$, ** to $p < 0.01$, *** to $p < 0.001$, and **** to $p < 0.0001$. Statistical analyses were performed on GraphPad Prism version 11.0.1 for Windows, GraphPad Software, San Diego, CA, USA, www.graphpad.com.

Scheme S1: Sugar structures

G0F	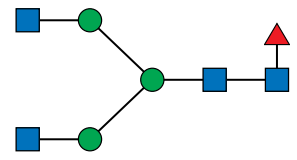		
G1F	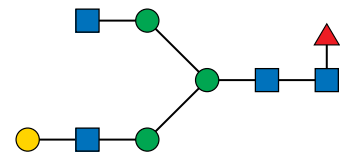	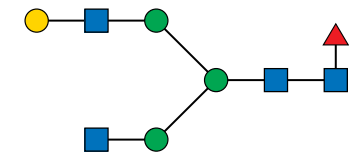	
-NAF	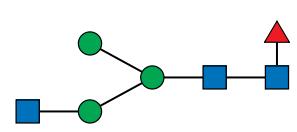	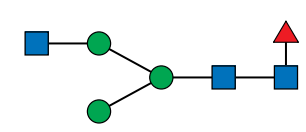	
Man5	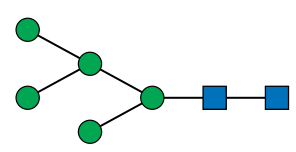		
Man6	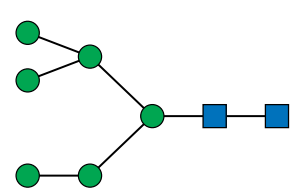		
G1	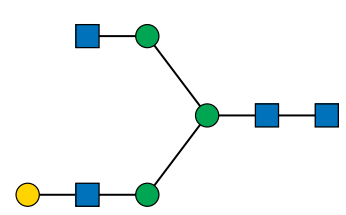	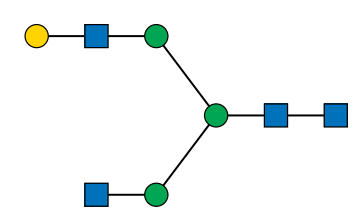	
H2N3F	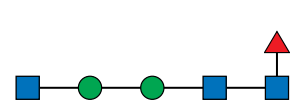	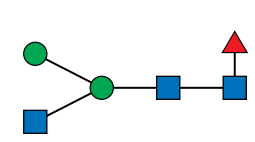	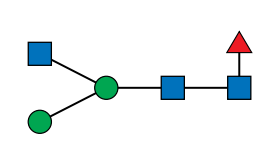
H4N3F	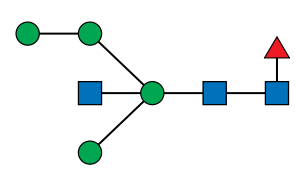		
Man7	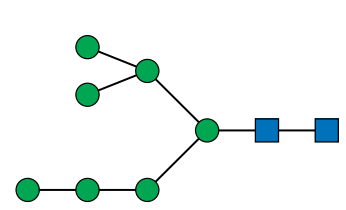	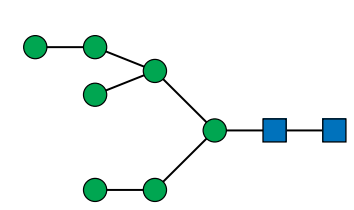	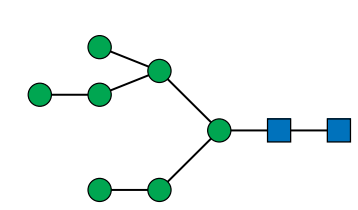
G0F + bi	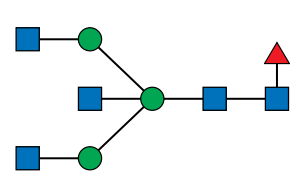		

G0	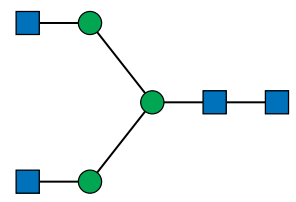
G2F	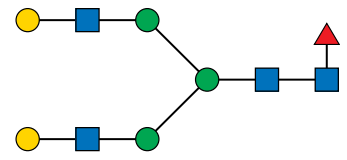
H3N2F	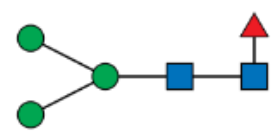
G2	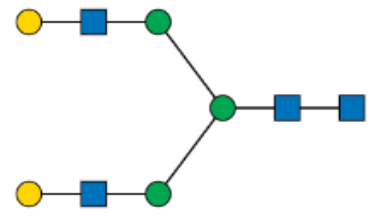

Structures drawn using https://glycotoolkit.com/Tools/GlycoGlyph/.

https://doi.org/10.1093/bioinformatics/btaa190
